# Harnessing Single-Cell RNA-Seq for Computational Drug Repurposing in Cancer Immunotherapy

**DOI:** 10.3390/ph18111769

**Published:** 2025-11-20

**Authors:** Olivia J. Cheng, T.T.T. Tran, Y. Ann Chen, Aik Choon Tan

**Affiliations:** 1 Department of Oncological Sciences, School of Medicine, University of Utah, Salt Lake City, UT 84112, USA; 2 Huntsman Cancer Institute, University of Utah, Salt Lake City, UT 84112, USA; 3 Division of Epidemiology, Department of Internal Medicine, School of Medicine, University of Utah, Salt Lake City, UT 84112, USA

**Keywords:** immunotherapy, immune checkpoint inhibitors, immune checkpoint inhibitors, combination therapies, single-cell RNA sequencing, tumor microenvironment

## Abstract

Immune checkpoint inhibitors (ICIs) have revolutionized cancer treatment and show notable success in some cancer types such as non-small cell lung cancer, melanoma and colorectal cancers, while they demonstrate relatively low response rate in others, such as esophageal cancers. Due to the heterogeneous nature of the tumor microenvironment and patient-to-patient variability, there remains a need to improve ICI response rates. Combining ICIs with therapies that can overcome resistance is a promising strategy. Compared to de novo drug development, drug repurposing offers a faster and more cost-effective approach to identifying such combination candidates. A variety of computational drug repurposing tools leverage genomics and/or transcriptomic data. As single-cell RNA sequencing (scRNA-seq) technology becomes available, it enables precise targeting of cancer-driving cellular components. In this review, we highlight current computational drug repurposing tools utilizing scRNA-seq data and demonstrate the application of two such tools, scDrug and scDrugPrio, on an esophageal squamous cell carcinoma dataset to identify potential drug candidates for combination with ICI therapy to enhance treatment response. scDrug focuses on predicting tumor cell-specific cytotoxicity, while scDrugPrio prioritizes drugs by reversing gene signatures associated with ICI non-responsiveness across diverse tumor microenvironment cell types. Together, this review underscores the importance of a multi-faceted approach in computational drug repurposing and highlights its potential for identifying drugs that enhance ICI treatment. Future work can expand the application of these strategies to multi-omics and spatial transcriptomics datasets, as well as personalized patient samples, to further refine drug repurposing involving ICI therapy.

## 1. Introduction

### 1.1. Immune Checkpoint Inhibitors: Efficacy and Resistance Mechanisms

Immune checkpoint inhibitors (ICIs), such as monoclonal antibodies targeting Programmed Cell Death Protein 1 (PD-1), Programmed Death-Ligand 1 (PD-L1) and Cytotoxic T-Lymphocyte-Associated Protein 4 (CTLA-4), have revolutionized cancer therapy by reactivating suppressed anti-tumor immune responses. In both clinical trials and real-world settings, cancers such as melanoma, non-small cell lung cancer (NSCLC), colorectal cancer, kidney cancer, bladder cancer, and head and neck cancer demonstrate relatively higher response rates to ICIs [1]. However, the overall average response remains substantially low, with considerable variation driven by cancer subtype, patient heterogeneity, and treatment context [1,2,3,4]. These cancers are sometimes referred to as “immune cold” tumors. Limited efficacy is largely attributed to the complexity and heterogeneity of tumors and their microenvironments, which give rise to a wide range of ICI resistance mechanisms. These include both tumor intrinsic factors, such as impaired antigen presentation, dysregulated signaling and metabolic pathways, and tumor-extrinsic mechanisms, such as the loss or dysfunction of effector immune populations, along with the enrichment of immunosuppressive populations and signaling pathways [5,6,7]. To circumvent these obstacles that dampen the efficacy of ICIs, ongoing research focuses on developing strategies that target and modulate these resistance pathways, often through combination therapies involving ICIs and other therapies [8,9,10].

### 1.2. Drug Repurposing to Enhance ICI Efficacy

Compared to de novo drug discovery, repurposing existing drugs approved for other indications offers a faster and more cost-effective path to clinical application, owing to their established safety profiles [11]. In the United States, drug repurposing is supported by streamlined development and approval process through the 505(b)(2) regulatory submission pathway. This pathway allows for changes such as new indications to previously approved drugs, partly based on established safety data, thereby reducing the need redundant preclinical and clinical studies [12,13,14]. Rather than replacing current immunotherapies, drug repurposing can also be leveraged to identify synergistic therapies to complement ICIs and improve response rates [8,9,15,16,17,18].

To overcome resistance, ongoing efforts have been focusing on identifying and testing drug combinations to enhance ICI treatment efficacy. In addition to dual ICIs, various drugs are also being explored in combination with ICIs to improve the efficacy of treatments [8,10,19,20,21]. Drugs targeting various cancer hallmarks for ICI resistance—such as metabolic modulators, hormone receptor inhibitors, immunomodulators, and anti-inflammatory agents, as well as microbial therapy—have been studied in preclinical studies, and these same agents, or others targeting similar pathways, are currently being evaluated in clinical trials [22,23,24,25,26,27] (Table 1).

**Table 1:** Selected clinical trials investigating the use of various therapies in combination with immune checkpoint inhibitors. CFH—Complement factor H.

Drug Type	Repurposed Drug	Tumor Intrinsic vs. Extrinsic Mechanisms	ClinicalTrials.gov Number (NCT)	Cancer Type	ICI	Phase	Status
Metabolic Modulators							
AMPK activator and mitochondrial complex I inhibitor	Metformin	Both [9,28,29]	NCT04414540	Head and Neck Cancer	Pembrolizumab	2	Active, not recruiting
			NCT03800602	Colorectal Cancer	Nivolumab	2	Completed
			NCT03618654	Head and Neck Cancer	Durvalumab	Early 1	Completed
			NCT03311308	Melanoma	Pembrolizumab	1	Recruiting
Statin	Lovastatin	Intrinsic [30,31]	NCT06636734	Head and Neck Cancer	Pembrolizumab	2	Recruiting
Hormone modulators							
Androgen Receptor Inhibitor	Darolutamide	Intrinsic [32]	NCT07016399	Triple-Negative Breast Cancer	Pembrolizumab	2	Not yet recruiting
Aromatase Inhibitors	Anastrozole, Letrozole, or Exemestane	Intrinsic [33]	NCT02648477	Triple-Negative or Hormone-Receptor Positive Breast Cancer	Pembrolizumab	2	Completed
Immunomodulators							
IDO1 inhibitor	BMS986205	Both [34]	NCT03854032	Head and Neck Cancer	Nivolumab	2	Active, not recruiting
	Epacadostat		NCT03358472	Head and Neck Cancer	Pembrolizumab	3	Active, not recruiting
			NCT03322540	Non-Small Cell Lung Cancer	Pembrolizumab	2	Completed
CSF1R inhibitor	Axatilimab	Extrinsic [35]	NCT07015853	Triple-Negative Breast Cancer	Pembrolizumab	2	Not yet recruiting
CXCR4 inhibitor	BL-8040	Extrinsic [36]	NCT02907099	Pancreatic Cancer	Pembrolizumab	2	Completed
Galectin-3 inhibitor	GB1211	Extrinsic [37]	NCT05913388	Melanoma, Head and Neck Cancer	Pembrolizumab	2	Recruiting
CFH inhibitor	GT103	Extrinsic (tumor-derived) [38]	NCT07017829	Non-Small Cell Lung Cancer	Pembrolizumab	2	Not yet recruiting
Cancer vaccine	IMA970A	Extrinsic [39]	NCT06218511	Hepatocellular carcinoma	Durvalumab	1	Recruiting
	p53MVA		NCT02432963	Solid Tumors	Pembrolizumab	1	Active, not recruiting
Live biotherapeutic products							
	Lactobacillus johnsonii	Extrinsic [40,41]	NCT06823323	Colorectal Cancer	Pembrolizumab	NA	Not yet recruiting
	CBM588 (*Clostridium butyricum*)	Both [41]	NCT06399419	Kidney Cancer	Nivolumab + Ipilimumab	1	Recruiting
	Microbial Ecosystem Therapeutic 4, MET4 (30 microbials)	Extrinsic [42]	NCT03686202	Solid Tumors	ICIs	1	Active, not recruiting
Anti-Inflammatory Agents/NSAIDs/COX inhibitors							
NSAID	Dicofenac	Intrinsic [9,43]	NCT06731270	Non-Small Cell Lung Cancer	Multiple	2	Recruiting
COX inhibitor	Aspirin	Both [9,44,45]	NCT02659384	Ovarian Cancer	Atezolizumab	2	Completed
			NCT03638297	Colorectal Cancer	a-PD-1	2	Recruiting
			NCT03396952	Melanoma	Pembrolizumab + Ipilimumab	2	Completed
COX inhibitor + platelet inhibitor	Clopidogrel/acetylsalicylic acid	Both [44,45,46]	NCT03245489	Head and Neck Cancer	Pembrolizumab	1	Completed
Kinase and Receptor Inhibitors							
FGFR4 inhibitor	Irpagratinib	Both [47]	NCT07010497	Hepatocellular Carcinoma	Atezolizumab + Bevacizumab	2	Not yet recruiting
mTOR inhibitor	nab-rapamycin (ABI-009)	Both [48,49]	NCT03190174	Multiple	Nivolumab	1/2	Completed
PI3K- α inhibitor	Alpelisib	Intrinsic [50]	NCT06545682	Breast Cancer and Melanoma	Pembrolizumab	1/2	Recruiting
Multi-target tyrosine kinase inhibitor	Lenvatinib	Both [51]	NCT07011849	Renal Cell Carcinoma	Pembrolizumab	2	Not yet recruiting
	Cabozantinib	Both [52]	NCT06900595	Adrenocortical Cancer	Cemiplimab	2	Not yet recruiting
	Cabozantinib	Extrinsic [53]	NCT03468218	Head and Neck Cancer	Pembrolizumab	2	Active, not recruiting
a-VEGFR2 antibody	Ramucirumab	Both [54]	NCT04120454	Non-Small Cell Lung Cancer	Pembrolizumab	2	Completed
VEGFR2 inhibitor	Anlotinib		NCT05218629	Pancreatic Cancer	a-PD-1	2	Recruiting
a-VEGF antibody	Bevacizumab		NCT03141684	Alveolar Soft Part Sarcoma	Atezolizumab	2	Active, not recruiting
a-TNF	Infliximab or Certolizumab	Both [55,56,57]	NCT03293784	Melanoma	Nivolumab + Ipilimumab	1	Completed
Anti-helminth drugs							
	Ivermectin	Both [58]	NCT05318469	Breast Cancer	Pembrolizumab	1/2	Recruiting

### 1.3. Computational Tools for Drug Repurposing

Computational drug repurposing offers an in silico alternative to traditional experimental approaches, allowing for rapid hypothesis generation and drug prioritization through the analysis of large-scale, high-throughput multi-omics data while requiring less labor, cost, and physical resources [11,18,59]. Several comprehensive reviews provide detailed overviews on the diverse computational approaches used in drug repurposing, including literature and text mining, docking, structural similarity, gene perturbation, etc. [60,61]. Specifically, drug repurposing and combination therapies have been explored as approaches to overcome resistance to ICIs by targeting various biological mechanisms and pathways [62]. In this review, we discuss how computational strategies that utilize single-cell RNA sequencing (scRNA-seq) data can be used to identify potential therapies that may enhance ICI response.

Reference databases are the key foundations for drug repurposing. The Kyoto Encyclopedia of Genes and Genomes (KEGG) [63] provides a reference of curated knowledge, integrating genes and genome, molecular functions and pathways, disease and drug-gene target information. In addition to such knowledge-based repositories, data-driven pharmacological perturbation resources provide empirical, cell-specific transcriptional responses to drug treatment. Many transcriptomics-based drug repurposing tools build upon the foundational concept introduced by the Connectivity Map (CMap)**,** which matches disease-associated gene expression signatures with drug-induced transcriptional responses [64]. CMap, along with complementary and expanded databases such as Drug Signatures Database (DSigDB), DrugSig, the Drug Repurposing Hub, the more recent L1000-based resources, and curated interaction databases like DrugBank and DGIdb (Table 2), serve as foundational references for several drug prediction tools and exploration platforms that leverage the concept of signature matching on disease-specific gene signatures generated from arrays or bulk RNA-seq, including iLINCS, DrInsight, L1000FWD and L1000CDS2 (Table 3).

**Table 2:** Key databases and libraries of drug-gene interactions and annotations.

Resources	Description	Website
DrugMAP [65]	A comprehensive database providing a molecular interaction atlas and pharmacological information for over 30,000 drugs and candidates, supporting drug discovery and AI-driven network analyses.	https://drugmap.idrblab.net/ (accessed on 21 August 2025)
L1000 [66]	A high-throughput, low-cost gene expression profiling method that enables large-scale mapping of cellular responses to genetic and chemical perturbations, expanding the Connectivity Map resource.	https://lincsproject.org/LINCS/tools (accessed on 21 August 2025)
DrugSig [67]	A manually curated database of drug response gene signatures from microarray data, designed to facilitate computational drug repositioning by providing comprehensive drug, gene, and target information.	http://biotechlab.fudan.edu.cn/database/drugsig (accessed on 21 August 2025)
The Drug Repurposing Hub [68]	A curated collection of over 4700 clinically tested compounds with detailed annotations, designed to enable systematic and large-scale drug repurposing efforts by providing resource for rapid identification of new therapeutic uses.	https://repo-hub.broadinstitute.org/repurposing (accessed on 21 August 2025)
DSigDB [69]	A manually curated database of drug and small molecule-related gene sets designed to integrate seamlessly with Gene Set Enrichment Analysis (GSEA), enabling researchers to analyze drug-induced gene expression changes and drug-target interactions.	https://dsigdb.tanlab.org/DSigDBv1.0/ (accessed on 21 August 2025)
ChEMBL [70,71]	A comprehensive database that curates and standardizes bioactivity, binding for over a million drug-like compounds and thousands of protein targets to support drug discovery and chemical biology research.	https://www.ebi.ac.uk/chembl/ (accessed on 21 August 2025)
DGIDB [72,73]	A comprehensive resource that integrates drug-gene interactions and potential druggable gene categories from multiple sources, enabling researchers to explore therapeutic targets for mutated genes and prioritize candidates for drug development.	https://dgidb.org/ (accessed on 21 August 2025)
DrugBank [74]	A comprehensive, searchable database combining detailed drug and target information to support drug discovery and pharmacological research.	https://go.drugbank.com/releases/latest (accessed on 21 August 2025)
CMap (Connectivity Map) [64]	A reference collection of gene-expression profiles from human cells treated with bioactive small molecules, enabling discovery of functional connections among drugs, diseases, and genetic perturbations.	https://clue.io/ (accessed on 21 August 2025)
Kyoto Encyclopedia of Genes and Genomes (KEGG) [63]	An encyclopedia of genes and genomes that integrates additional information regarding molecular functions and pathways, drugs and disease.	http://www.genome.jp/kegg/ (accessed on 14 October 2025)

**Table 3:** Computational drug prediction tools using gene expression signatures.

Tool	Platform	Description	Website
RepurposeDrugs [75]	Web-based	A web platform that combines a comprehensive drug–indication database with machine learning to predict the approval potential of mono- and combination therapies for new disease indications.	https://repurposedrugs.aittokallio.group/ (accessed on 21 August 2025)
TxGNN [76]	Web-based	A graph-based AI model for zero-shot drug repurposing that predicts new drug–disease associations, including for diseases with no known treatments, using interpretable reasoning over a medical knowledge graph.	http://txgnn.org/ (accessed on 21 August 2025)
DRIE [77]	R	A framework to identify candidate drugs for cancer based on inhibition effect on disease-specific gene regulatory network of KEGG pathways.	
SigCom LINCS [78]	Web-based	A webserver offering over a million searchable gene expression signatures from LINCS, GTEx, and GEO to support drug and target discovery through signature similarity and metadata analysis	https://maayanlab.cloud/sigcom-lincs/#/SignatureSearch/UpDown (accessed on 21 August 2025)
DRviaSPCN [79]	R	A tool for cancer drug repurposing that prioritizes candidate drugs by analyzing drug-induced sub-pathway crosstalk networks and their influence on tumor pathways.	
iLINCS [80]	Web-based	A web platform that integrates large-scale omics data and tools for analysis, visualization, mechanism of action studies, and drug repositioning without requiring programming skills.	http://ilincs2018.ilincs.org/ilincs/ (accessed on 21 August 2025)
DrInsight [81]	Python	A method for drug repurposing that uses genome-wide concordantly expressed genes to improve disease-drug matching, outperforming existing methods and enabling comprehensive drug-target network analysis.	
SAveRUNNER [82]	R	A network-based tool for drug repurposing that predicts new drug-disease associations by analyzing the proximity of drug targets and disease proteins in the human interactome	
DREIMT [83]	Web-based	A web tool that prioritizes immunomodulatory drugs targeting up to 70 immune cell subtypes by integrating thousands of drug profiles and immune gene expression signatures, helping to identify potential therapies based on user-provided gene expression data.	https://dreimt.org/ (accessed on 21 August 2025)
DrugCell [84]	Python	An interpretable deep learning model that predicts cancer drug responses by integrating tumor genotypes and drug structures, enabling accurate therapy predictions and the design of synergistic drug combinations.	
L1000FWD [85]	Web-based	An interactive web tool for visualizing and exploring over 16,000 drug- and small-molecule-induced gene expression signatures to aid in understanding drug mechanisms of action and discovering novel compound functions.	https://maayanlab.cloud/l1000fwd/ (accessed on 21 August 2025)
L1000CDS2 [86]	Web-based	A web-based search engine that prioritizes small molecules predicted to mimic or reverse gene expression signatures, enabling drug prediction and target identification from the LINCS L1000 dataset.	https://maayanlab.cloud/L1000CDS2/#/index (accessed on 21 August 2025)

Since its breakthrough development in 2009 and broader adoption in 2017, scRNA-seq has rapidly expanded in use [87,88,89], especially in cancer research to understand the tumor heterogeneity and tumor-immune microenvironment. Consequently, there is a growing number of computational drug repurposing tools and methods that aim to harness the benefits from scRNA-seq and the rapidly expanding availability of single-cell datasets (Table 4). Compared to bulk RNA-seq, scRNA-seq offers transcriptomic profiling at increased cellular resolution, allowing for granular analysis using gene expression at the level of individual cell types and states. This is particularly informative in cancer research, where tumors often display marked heterogeneity both within and across patients, including key disease contributing factors that would otherwise be masked in bulk transcriptomic data due to their rarity and low abundance [90,91]. Specifically, scRNA-seq provides insights into the tumor microenvironment and clonal diversity—a complex network of interactions among cancer and non-cancer cells that plays an important role in tumor development, immune evasion, and cancer therapy response and resistance [89,91,92,93,94,95,96]. While earlier tools using bulk transcriptomic input for signature matching can be adapted to use the scRNA-derived differentially expressed gene (DEG) profile, newer tools and frameworks designed to use scRNA-seq data more directly have also been released, including scDR, scDEAL, scDrug, ASGARD, scDrug+, DrugReSC, scTherapy, scDrugPrio, and retriever (Table 4). These high-resolution, targeted approaches allow researchers to target the specific cell types and states, improving the precision and biological relevance of drug predictions.

Most single-cell-informed drug repurposing tools, including scTherapy, DrugReSC, and scDrug, utilize the drug response profile from resources like LINCS-L1000, GDSC, or PRISM, which are predominantly derived from cancer cell lines. As a result, these tools primarily focus on identifying drugs that act directly on malignant cell populations [97,98,99]. In contrast, tools such as drexml and scDrugPrio use drug-target databases like DrugBank. These tools are not cancer-specific and support drug prediction for non-malignant or diverse cell types, including immune cells [100,101]. While ASGARD utilizes drug response data from LINCS-L1000, the original publication demonstrated its capacity in drug repurposing for multiple cancers as well as COVID-19 [102]. Furthermore, while most tools provide monotherapy predictions, scTherapy, ComboSC, and scDrug support combination therapy prediction for optimal treatment by targeting multiple pathways. In addition, these tools also differ in their computational approaches. Tools like scTherapy, scDEAL, and drexml leverage machine learning models, including deep learning and explainable machine learning, while others such as scDrugPrio and ComboSC rely on network-based strategies to capture cellular interactions [100,103]. Together, these tools highlight the growing potential of single-cell transcriptomics to inform precision drug repurposing across a range of diseases, enabling more targeted therapeutic strategies to address interpatient and cellular-level heterogeneity in complex conditions such as cancer (Table 4).

**Table 4:** Computational drug repurposing tools and pipelines utilizing single-cell data input.

Tool	Platform	Description	Cancer-Focused?
retriever [104]	R	A tool that identifies disease-specific transcriptional drug response profiles from LINCS-L1000 data to predict effective drug combinations for personalized cancer treatment	Yes
scDrugPrio [100]	R	A framework that uses scRNA-seq data and drug-target information to build disease network models for prioritizing and ranking drugs in immune-mediated inflammatory diseases	No
drexml [101]	Python	A command line tool and package for data-driven drug repurposing that combines mechanistic signal transduction modeling with explainable machine learning to characterize disease-specific regulatory networks and rank drug targets based on their functional impact on disease signaling.	No
SuperFeat [105]	Python	An artificial neural network–based framework that learns and scores canonical cellular features from single-cell RNA-seq data to identify disease progression markers and potential drug targets.	Yes Note: Can perform cellular status./feature scoring on all cell types but drug search focuses on malignant cells.
scTherapy [99]	R	A machine learning framework that uses single-cell transcriptomics to prioritize personalized multi-target drug combinations selectively targeting cancer cells while sparing normal cells.	Yes
DrugReSC [98]	R	A method that leverages single-cell RNA-seq data to identify drugs targeting disease-critical cell subpopulations through transcriptional relationship modeling.	Yes Note: Identifies disease-central cells (not restricted to tumor) but uses LINCS for drug matching.
scDrug+ [106]	Python	An integrated pipeline combining single-cell transcriptomics and molecular structure analysis to predict drug responses, including for novel drugs, enabling precision medicine.	Yes
ComboSC [103]	R	A pipeline that uses single-cell transcriptomes and bipartite graph optimization to predict personalized synergistic drug combinations, enhancing precision cancer immunotherapy.	No
ASGARD [102]	R	A pipeline that accounts for cellular heterogeneity to improve personalized drug recommendations, outperforming bulk-based methods	No
scDrug [97]	Python	An integrated workflow that streamlines scRNA-seq analysis and drug response prediction to facilitate tumor subpopulation identification and drug repurposing.	Yes
DREEP [107]	R	A tool that predicts drug sensitivity at the single-cell level using transcriptomic data and pharmacogenomic references to guide personalized cancer treatment and drug repurposing.	Yes
scDR [108]	R	A method that predicts drug response at single-cell resolution by integrating drug-response genes with scRNA-seq data, enabling prognosis prediction and exploration of drug resistance mechanisms	Yes
scDEAL [109]	Python	A deep transfer learning framework that predicts cancer drug responses at the single-cell level by integrating bulk RNA-seq data with scRNA-seq and offers interpretable gene signatures linked to drug resistance.	Yes
Beyondcell [110]	R	A method that identifies tumor subpopulations with distinct drug responses from scRNA-seq data and ranks cancer-specific treatments using drug signature enrichment.	Yes

## 2. Case Study: Two Computational Strategies for Identifying Drug Repurposing Candidates to Enhance ICI Response

**Figure 1:**
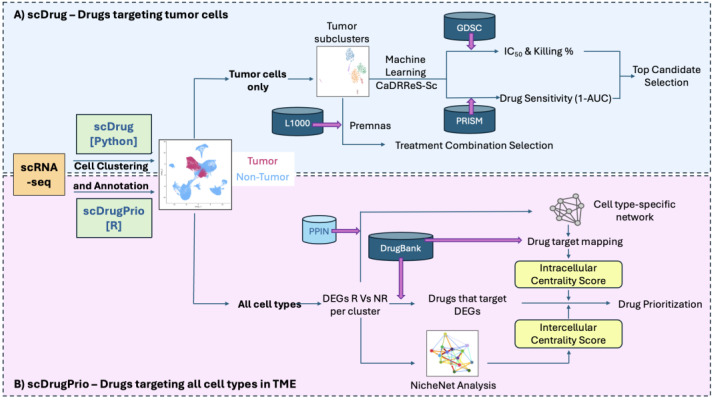
Summary workflows of drug repurposing analysis using scRNA-seq data of ESCC samples from treatment responders and non-responders with (**A**) scDrug and (**B**) scDrugPrio. CaDRReS-Sc—Cancer Drug Response prediction using a Recommender System-Single cell. GDSC—Genomics of Drug Sensitivity in Cancer. IC50—Half maximal inhibitory concentration. AUC—Area under the curve. TME—Tumor microenvironment. DEG—Differentially expressed genes. R—Responder. NR—Non-responder. PPIN—Protein–protein interaction network.

In this section, we review the overall workflows and application of two computational tools listed in Table 4 that utilize scRNA-seq as input data, scDrug and scDrugPrio. These two workflows represent distinct methodological approaches: scDrug aims to selectively target tumor cells, whereas scDrugPrio aims to counteract disease-associated transcriptomic signatures across all cell types (Figure 1).

To demonstrate their application in the context of ICI response, we applied both frameworks to a publicly available scRNA-seq dataset derived from esophageal squamous cell carcinoma (ESCC) patients. Compared to the more ICI-responsive cancers such as melanoma, the objective response rate (ORR) to anti-PD-(L)1 monotherapy in ESCC is relatively low (~10–20% vs. ~30–40%) [1,111]. Furthermore, ESCC remains underrepresented in immunotherapy research, receiving less attention in clinical trials compared to more common cancers like melanoma and NSCLC, highlighting a critical opportunity for novel insights [112]. The dataset was obtained from OMIX, China National Center for Bioinformation/Beijing Institute of Genomics, Chinese Academy of Sciences (accession number: OMIX005710) [113]. Patients in the cohort received neoadjuvant chemo-immunotherapy (NAT), which included rislelizumab or camrelizumab, in combination with carboplatin or nedaplatin, and albumin-bound paclitaxel. To illustrate the utility of the computational methods, we used the baseline (pre-treatment) scRNA-seq tumor samples and associated clinical outcome data of this cohort. Treatment response was determined in the original study based on the presence of viable residual tumor cells (VRTCs) in the resected tumor and lymph nodes. VRTCs ≥ 1% and ≤ 10% was considered as major pathological response (MPR), ≤ 1% was defined as pathological complete response (pCR) and >10% was defined as incomplete pathological response (IPR). In our study, pCR and MPR were grouped as response, and IPR was considered as no response. While traditional applications of drug repurposing tools focus on identifying therapies for a given disease or cancer, here we adapt the approach to enhance ICI response by treating non-responder samples as the “disease” condition.

### 2.1. Method 1: scDrug—Targeting Tumor Cells

In 2023, Hsieh et al. introduced scDrug (https://github.com/ailabstw/scDrug, accessed on 13 October 2025), a Python based one-step computational pipeline designed to link single-cell transcriptomics with drug response prediction, particularly focusing on tumor cell subpopulations. scDrug integrates clustering, functional annotation, and pharmacogenomic modeling into a single framework.

The workflow begins with scRNA-seq data processing and analysis using SCANPY [114] (Figure 1). For quality control, cells with less than 200 genes and more than 30% mitochondrial content were excluded. Normalization was performed as described in https://scanpy.readthedocs.io/en/stable/tutorials/basics/clustering.html (accessed on 16 October 2025) and batch integration was performed using Harmony algorithm [14]. The scDrug pipeline identifies tumor clusters from the scRNA-seq dataset through analysis with the Louvain algorithm for unsupervised clustering and resolution was determined empirically via silhouette-based optimization using repeated subsampling. scMATCH was used for cell type annotation using the FANTOM5 reference dataset [97,115,116,117]. Tumor populations are then selected, re-clustered and used as the input for drug response prediction using public pharmacogenomic datasets with two independent predictive models. One prediction approach utilizes CaDRReS-Sc [118], a machine learning framework trained on the GDSC dataset (includes drug response data for 1047 cancer cell lines across 266 anti-cancer drugs) and the PRISM repurposing dataset (covering approximately 1448 drugs across 480 cell lines, quantified by 1 − AUC) to predict drug responses [119,120]. The second approach employs the Premnas framework, which leverages LINCS L1000 perturbation signature data to predict potential treatment combinations [121].

The aim of scDrug is to identify drugs and drug combinations that target and kill tumor clusters identified using scRNA-seq data. To tailor this to enhance ICI response, we applied the scDrug pipeline to the pre-treatment ESCC tumor samples from patients who later were classified as non-responders to neoadjuvant chemo-immunotherapy. The goal was to identify drug candidates that could potentially improve cancer killing in these treatment-resistant cases.

As a demonstration, we present one of the drug response analyses from scDrug workflow using the GDSC dataset as a reference. After selecting tumor populations, scDrug re-clustered them into seven distinct tumor subclusters. Drug response (IC_50_ and estimated killing percentage) was predicted for each cluster across all 171 compounds from the GDSC reference dataset.

**Figure 2:**
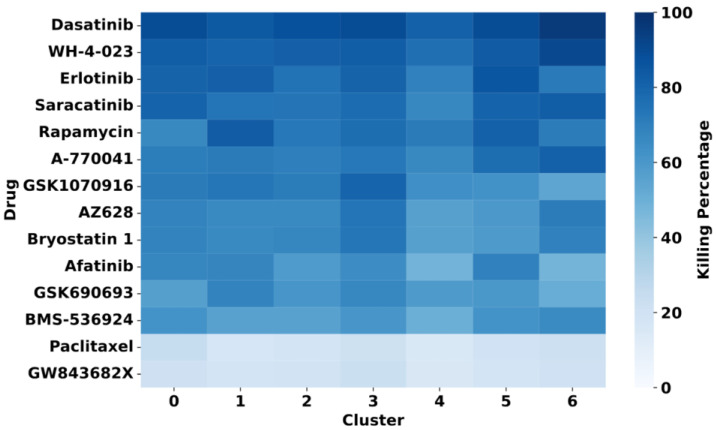
Top drugs from the GDSC database showing more than 60% predicted killing in four or more tumor subclusters, based on scDrug analysis (Paclitaxel and GW843682X (with <20% average killing) are included as comparators).

Among the 171 drugs analyzed, 12 compounds—including EGFR inhibitors, Src family kinase inhibitors, and metabolic modulators—exhibited more than 60% of killing in four or more tumor subclusters (Figure 2). Notably, the Src family kinase inhibitor Dasatinib showed the highest predicted efficacy, exceeding 80% predicted killing in all seven tumor clusters.

In ESCC, dasatinib has been shown to enhance cisplatin sensitivity by modulating key resistance pathways, such as PI3K/AKT and STAT3, and downregulating resistance-associated molecules such as ERCC1 and BRCA1 [122], supporting its therapeutic relevance in this tumor type. Specifically in the context of ICI treatments, Dasatinib has demonstrated synergistic effects when combined with anti-PD-L1 in breast cancer models, reducing cancer cell viability, colony formation, and invasion [123]. Moreover, Dasatinib combined with anti-PD-1 has been shown to reduce tumor burden and improve overall survival in preclinical models of Philadelphia chromosome-positive acute lymphoblastic leukemia, colorectal cancer, and non-small-cell lung cancer [124,125,126]. These reports support that other Src family kinase inhibitors with high predicted efficacy, such as saracatinib and WH-4-023, may also warrant further investigation as potential ICI-enhancing agents.

Additional candidates identified by scDrug include rapamycin, an mTOR inhibitor that has been reported to enhance anti-PD-1 efficacy in colorectal cancer models [127] and has been investigated clinically for its efficacy in combination with anti-PD-1 (Table 1). Similarly, epidermal growth factor receptor (EGFR) inhibitors such as erlotinib have been evaluated in early-phase clinical trials (NCT02039674, NCT02013219) in combination with pembrolizumab or atezolizumab, with preliminary results demonstrating partial responses in some patients, while no complete responses were observed [128,129].

Together, these examples serve as a proof-of-concept that top candidates identified by scDrug analysis of ESCC non-responder data align with drugs that have either demonstrated or are emerging as potentials to enhance ICI response in preclinical or clinical settings. This supports the feasibility and relevance of scDrug as a strategy for selecting promising drug candidates for ICI-based combination therapy.

Despite this utility, there are important limitations to consider. First, drug response predictions are constrained by the coverage of drug sensitivity data available in the reference databases, which may not comprehensively represent all clinically relevant compounds. Second, the module for treatment combination selection depends heavily on the availability of drug-induced gene expression profiles from the LINCS L1000 dataset. The 2017 release of the LINCS lacked ESCC cell line data entirely. Although the updated release expanded coverage to 98 cancer cell lines [130], including the ESCC cell line KYSE30.311, key dose–response information required for combination analysis remains unavailable. As a result, treatment combination prediction was not feasible for this dataset, underscoring the limitations of applying scDrug to less well-characterized cancer types. Thirdly, since scDrug focuses primarily on tumor cell populations, tumor-extrinsic factors within the tumor microenvironment—such as immune cell and stromal interactions—that are known to influence treatment outcomes are not accounted for.

### 2.2. Method 2: scDrugPrio—Targeting All Cell Types

Published in 2024, scDrugPrio (available as an R package version 1.0.0; https://github.com/SDTC-CPMed/scDrugPrio, accessed on 21 August 2025) is a network-based drug repurposing tool originally developed for inflammatory disease datasets. It identifies compounds from DrugBank [75] that may counteract cell-type-specific disease-associated gene signatures based on DEGs in each cell cluster between disease state and control. scDrugPrio then ranks the compounds based on proximity between drug targets and DEGs in protein–protein interaction network (PPIN) and centrality of drug targets across various disease-central cell populations.

The pipeline begins with preprocessing, clustering, and annotation of scRNA-seq data using Seurat [131] (Figure 1). Quality control is performed for each individual patient dataset, excluding cells with fewer than 200 detected genes (nFeature_RNA < 200). Mitochondrial content is capped at 30%. Normalization and integration follow the publicly available Seurat vignette (https://satijalab.org/seurat/articles/integration_introduction; accessed on 13 May 2025). Disease-associated differential gene signature for each cell type is identified using the FindMarkers function of Seurat.

To assess intercellular centrality score for each cluster, scDrugPrio integrates cell–cell interaction analysis via NicheNet [132], allowing for identification of cell types that are more influential in the disease state. Within each cell type, drugs are assigned intracellular centrality score based on the network centrality of their targets in the disease-associated subnetworks. The final drug ranking is based on a composite score of intra-and intercellular centralities for each drug across cell types, with intercellular centrality weighed more heavily. Specifically, the compound score is calculated as the sum of a drug’s intercellular centrality plus 0.1 times the sum of its intracellular centralities across all cell types. By combining these layers of information, scDrugPrio provides a prioritization framework that outputs a ranked list of drug candidates most likely to modulate the observed disease-associated gene signatures in disease-driving cell populations.

**Figure 3:**
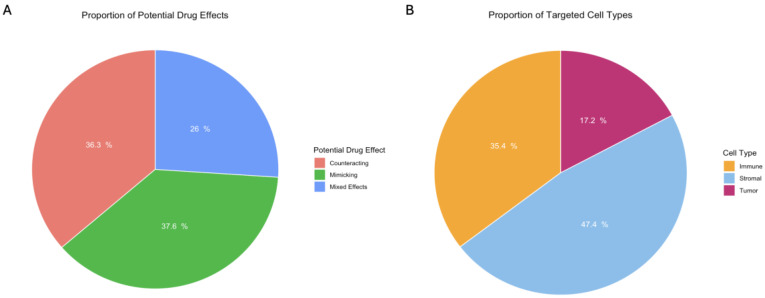
Proportion of (**A**) potential drug effects and (**B**) targeted cell types among the 622 entries in the unranked scDrugPrio list, comprising 129 unique drug candidates. Each entry represents a drug–cell type pair, where individual drugs may appear multiple times across different targeted cell types.

To demonstrate how scDrugPrio can be applied to identify drug candidates to enhance ICI response, we analyzed the ESCC pre-treatment samples from both responders and non-responders. scDrugPrio filters drugs from DrugBank based on network distance between cell type-specific DEGs and drugs targets in the PPIN for each cell type-drug pair, selecting only drugs with targets significantly closed to DEGs and frequently target DEGs directly. Using the resulting unranked list of 129 unique candidate drugs from 622 cell type-drug pairs, manual evaluation of each drug’s mechanism of actions allows for the identification of compounds that may counteract or mimic the gene expression patterns associated with non-responsiveness in each cell type (Figure 3). This preliminary drug set offers a rich starting point for hypothesis generation and biological interpretation.

Out of the 129 unique drug candidates, 60 of them are counteracting upregulated genes associated with no response. Using these 60 drugs as input into DrugEnrichr [133,134,135], 19 Drug Repurposing Hub mechanisms of action (MOA) are significantly enriched (Appendix A Table A1). The most significant MOA, cyclooxygenase (COX) inhibitor, is represented by six scDrugPrio predicted drugs. While the effects of COX inhibitors demonstrate mixed results in their effect on ICI response and outcome in various cancer types and patient cohorts [136,137,138,139,140], several are being evaluated in current clinical trials (Table 1). Additional enriched mechanisms include inhibitors of KIT, PDGFR, and VEGFR, which are shared targets of lenvatinib and dasatinib. Lenvatinib is being investigated in an ongoing clinical trial (Table 1), and a retrospective clinical study has demonstrated its concurrent use with ICI associates with improved survival compared to sequential administration of treatments [141]. Dasatinib has been shown to potentiate a-CTLA-4 treatment in an in vivo mouse, supporting their use in combination with ICI treatments [142].

**Figure 4:**
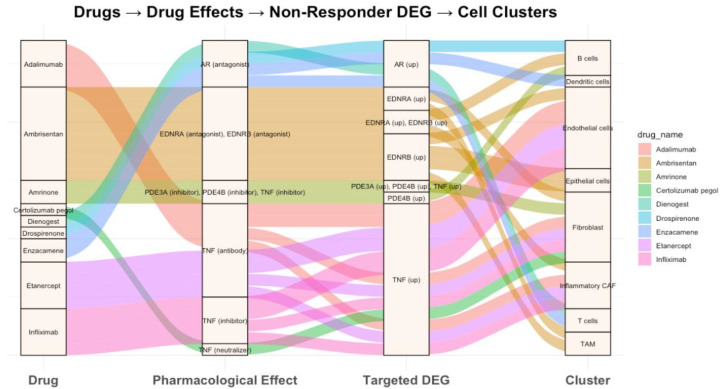
Selected drugs from scDrugPrio results on ESCC dataset and their corresponding pharmacological effects and targeted cell clusters. Colored flows represent each drug’s pathway from effect to gene target to cell cluster.

For individual drugs, several of the top hits aligned with previously published reports on their use in modulating ICI response, supporting the utility of scDrugPrio for drug repurposing to enhance ICI response. For example, several androgen receptor (AR) antagonists—including drospirenone, dienogest, and enzacamene—were identified and listed (Figure 4, Appendix A Table A2), consistent with approach to counteract the upregulated AR expression observed in non-responders in the ESCC cohort. Prior in vitro and in vivo studies in mouse models of hepatocellular carcinoma and prostate cancer have demonstrated that AR overexpression negatively attenuates anti-PD-L1, and that AR and PD-L1 dual blockade enhances the overall survival and T cell function [23,24]. Tumor necrosis factor (TNF) inhibitors and neutralizing antibodies, such as infliximab and certolizumab, were also identified by scDrugPrio (Figure 4, Appendix A Table A2). Both have been evaluated in a clinical trial for their potential to enhance ICI efficacy (NCT03293784, Table 1). On the other hand, Endothelin blocking has also been shown to potentiate responses to anti-CTLA4 and anti-PD1 therapies in a breast cancer model [143] and is also currently being investigated in a clinical trial (NCT07016399, Table 1). Notably, an endothelin receptor antagonist ambrisentan, which has been reported to inhibit cancer progression [144], was also among the scDrugPrio suggested candidates (Figure 4, Appendix A Table A2). Together, these examples highlight the potential of scDrugPrio to identify biologically relevant drug candidates for ICI efficacy enhancement.

Beyond identifying drugs that may help overcome resistance to ICI, the results from scDrugPrio can also be used to help flag compounds that may exacerbate non-responsiveness from the “disease” mimicking drugs in the unranked drug list from scDrugPrio analysis. For example, IL1R1 was downregulated in tumor-associated macrophages, mast cells, and dendritic cells in the non-responder group. While IL1R1 perturbation has been shown to sensitize resistant tumors in a lung cancer model, myeloid-specific ablation of IL1R1 can promote colorectal cancer progression [145,146]. Thus, targeting IL1R1 with inhibitory drugs like Anakinra might worsen ICI resistance and may not be applicable in the context of ESCC. Similarly, since AR expression levels was elevated in non-responders, use of an AR agonist Methyltestosterone would be counteractive and worsen response to treatment. These examples illustrate how scDrugPrio, by identifying compounds that mimic the gene expression signatures of non-responsiveness, can also help avoid compounds that may inadvertently worsen patient outcomes in a case-specific manner.

Like scDrug, the performance of scDrugPrio is also limited by the scope of drug information available in DrugBank. Most compounds are annotated primarily based on their original intended targets, while potential off-target effects—such as modulation of secondary genes or pathways—may not be captured in current annotations [9,17,147]. These unannotated effects may offer new therapeutic opportunities, particularly in the context of drug repurposing, but may be overlooked due to the limited extent of database annotations. Similarly, undocumented off-target effects may exacerbate disease or side effects, warranting future investigations into the toxicities of the predicted drug candidates.

## 3. Conclusions and Lessons Learned

The ability of several drug candidates identified by scDrug and scDrugPrio to enhance ICI efficacy is supported by existing preclinical and clinical evidence involving either the same or mechanistically related compounds. This highlights the potential of applying these computational drug repurposing tools to scRNA-seq datasets to uncover strategies to circumvent ICI treatment resistance. Although many of the identified drugs have been studied in cancer types other than ESCC, their prior use in combination with ICIs suggests the broader applicability and relevance of this approach. Despite the support of these predicted candidates from previous studies in their clinical and biological relevance in use with ICIs, in vitro and in vivo experiments are required to validate their capacity in enhancing ICI response.

**Figure 5:**
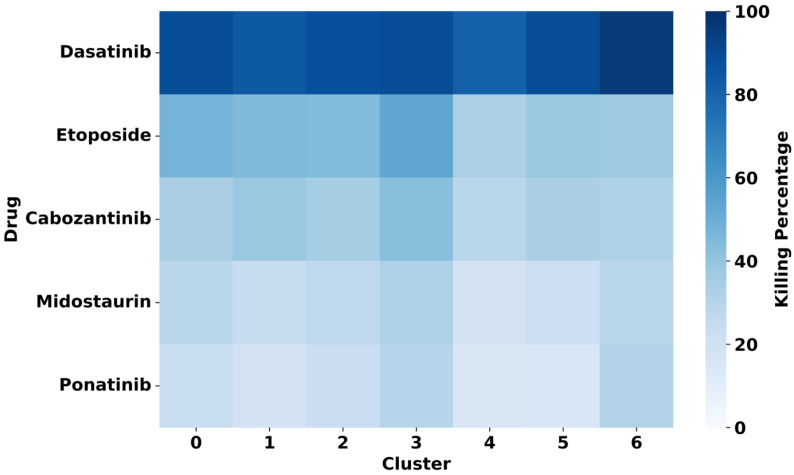
Predicted killing percentages across tumor subclusters for overlapped drugs between scDrug (GDSC) and scDrugPrio results.

Although scDrug and scDrugPrio employ distinct drug repurposing approaches and rely on reference databases with limited overlap in drug coverage, five drugs from the GDSC dataset were also identified in scDrugPrio: cabozantinib, dasatinib, etoposide, midostaurin, and ponatinib. Among these, dasatinib was ranked as the top candidate in scDrug based on its high predicted killing percentage across all tumor subclusters. The remaining four drugs, while exhibiting low predicted cytotoxicity in scDrug (Figure 5), may act on non-tumor components of the tumor microenvironment in non-responders, such as immune and non-malignant stromal cells, based on the scDrugPrio results (Table 5).

**Figure 6:**
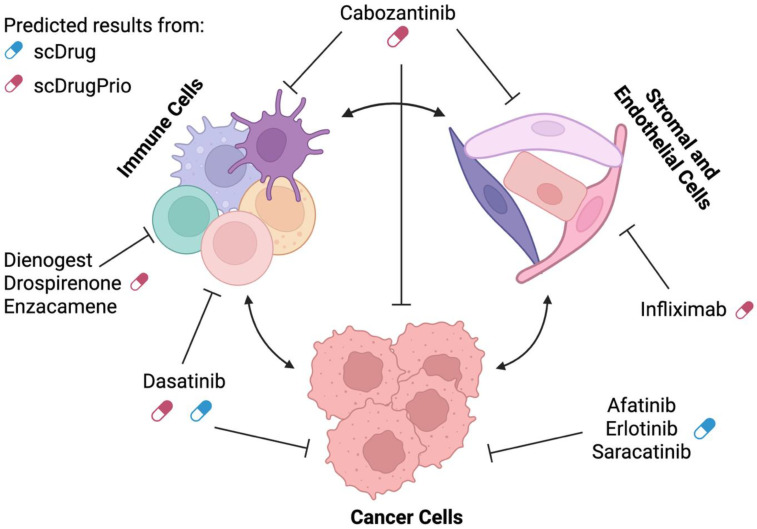
Selected drug candidates from scDrug and scDrugPrio predictions targeting tumor microenvironment components. This schematic illustrates the cellular composition of the tumor microenvironment, including cancer cells, immune cells, and stromal and endothelial cells. Predicted drug candidates identified by two drug repurposing tools—scDrug and scDrugPrio—are mapped to their respective cellular targets. Created in BioRender. CHENG, O. (2025) https://BioRender.com/lcev1pu (accessed on 9 September 2025).

This limited overlap highlights the complementary nature of the two tools, although each captures different aspects of tumor biology and therapeutic potentials through distinct strategies (Figure 6). scDrug integrates single-cell data into drug screening and focuses on cytotoxicity predictions on tumor populations, whereas scDrugPrio integrates single-cell data with network-based drug-gene interaction to reverse gene signatures associated with non-responsiveness across all cell types in the tumor microenvironment. Importantly, each tool has its limitations: scDrug does not account for non-tumor compartments, potentially overlooking critical interactions in the tumor microenvironment, while scDrugPrio lacks pharmacodynamic information, which is essential for understanding how different drugs modulate different cell populations. This means that even if a drug theoretically targets disease-associated gene expression in a given cell population, it may not be effective due to factors like drug uptake, metabolism, or resistance mechanisms specific to that cell type. However, taken together, these tools offer complementary insights for drug prioritization.

These workflow examples support a multi-tool, integrative approach for computational drug repurposing. Combining strategies used in scDrug and scDrugPrio enables the identification of drug candidates that target both tumor-intrinsic and -extrinsic hallmarks of ICI resistance while also incorporating drug response data. Such a combined approach may provide a more comprehensive and robust strategy for selecting drug candidates to enhance ICI treatment response. While computational approaches offer a time- and cost-effective means of drug screening, experimental validation of the results remains essential.

To further expand this application of computational drug repurposing, future work could apply these methods to multi-omics and spatial transcriptomics datasets or individual patient samples for personalized treatment design. Additionally, beyond enhancing efficacy, some of these approaches may be adapted to address other aspects of ICI therapy, such as mitigating immune-related adverse events, and could also be applied to other types of immunotherapies, including adoptive cell therapies.

## Figures and Tables

**Table 5:** Overlapped drugs with scDrug (GDSC) and their corresponding DrugBank information from scDrugPrio analysis of ESCC patient dataset.

Drug	DrugBank ID	Pharmacological Effect	Targeted Upregulated DEGs	Cluster
Dasatinib	DB01254	ABL1 (multitarget), SRC (multitarget), EPHA2 (antagonist), LCK (multitarget), YES1 (inhibitor), KIT (antagonist), PDGFRB (antagonist), STAT5B (inhibitor), ABL2 (multitarget), FYN (multitarget)	PDGFRB	T cells Endothelial cells Epithelial cells
Cabozantinib	DB08875	MET (antagonist), KDR (antagonist), RET (antagonist)	KDR	Mast cells Endothelial cells
Etoposide	DB00773	TOP2A (inhibitor), TOP2B (inhibitor)	TOP2A	T cells Fibroblast Endothelial cells Mast cells
Midostaurin	DB06595	PRKCA (antagonist, inhibitor), KDR (antagonist, inhibitor), KIT (antagonist, inhibitor), PDGFRA (antagonist, inhibitor), PDGFRB (antagonist, inhibitor), FLT3 (antagonist, inhibitor)	PDGFRA, FLT3	Tumor epithelial cells Endothelial cells
Ponatinib	DB08901	ABL1 (inhibitor), BCR (inhibitor), KIT (inhibitor), RET (inhibitor), TEK (inhibitor), FLT3 (inhibitor), FGFR1 (inhibitor), FGFR2 (inhibitor), FGFR3 (inhibitor), FGFR4 (inhibitor), LCK (inhibitor), SRC (inhibitor), LYN (inhibitor), KDR (inhibitor), PDGFRA (inhibitor)	KIT, PDGFRA	Cycling/proliferating epithelial cells
			LYN, KDR	Cancer-associated fibroblast (CAF)
			FGFR3, KDR, PDGFRA	Mast cells
			FLT3, KDR, PDGFRA	Epithelial cells
			FLT3, KDR, PDGFRA	Endothelial cells
			FLT3, KDR, PDGFRA	B cells

## Data Availability

All datasets used in the case studies are publicly available and cited within the manuscript.

## Abbreviations

The following abbreviations are used in this manuscript:

AUC	Area under the curve
AR	Androgen receptor
CaDRReS-Sc	Cancer Drug Response prediction using a Recommender System-Single cell
CAF	Cancer-associated fibroblast
GDSC	Genomics of Drug Sensitivity in Cancer
CFH	Complement factor H
CTLA-4	Cytotoxic T-Lymphocyte-Associated Protein 4
DEG	Differentially expressed genes
EGFR	Epidermal growth factor receptor
ESCC	Esophageal squamous cell carcinoma
IC50	Half maximal inhibitory concentration
ICI	Immune checkpoint inhibitors
NAT	Neoadjuvant chemo-immunotherapy
NR	Non-responder
NSCLC	Non-small cell lung cancer
ORR	Objective response rate
scRNA-seq	Single-cell RNA sequencing
TME	Tumor microenvironment
TNF	Tumor necrosis factor
PD-1	Programmed Cell Death Protein 1
PD-L1	Programmed Death-Ligand 1
PPIN	Protein–protein interaction network
R	Responder

## Appendix A

**Table A1:** Significant mechanisms of action associated with scDrugPrio-predicted drugs counteracting DEGs linked to non-response in ESCC patients, identified via DrugEnrichR enrichment analysis. Adjusted *p*-values were rounded to four decimal places.

Drug Repurpose Hub Mechanisms of Action	Overlap	Adjusted *p*-Value	Associated Drugs
Cyclooxygenase inhibitor	5/75	0.0001	Ketoprofen Propacetamol Bromfenac Nabumetone Mefenamic acid
KIT inhibitor	2/16	0.0114	Dasatinib Lenvatinib
PDGFR tyrosine kinase receptor inhibitor	2/20	0.0119	Dasatinib Lenvatinib
VEGFR inhibitor	2/29	0.0188	Lenvatinib Cabozantinib
Phosphodiesterase inhibitor	2/40	0.0282	Amrinone theobromine
Prostaglandin inhibitor	1/7	0.0367	Grapiprant
PKC inhibitor	1/9	0.0367	Dequalinium
Endothelin receptor antagonist	1/8	0.0367	Ambrisentan
CC chemokine receptor antagonist	1/7	0.0367	Plerixafor
RET tyrosine kinase inhibitor	1/7	0.0367	Cabozantinib
Tyrosine kinase inhibitor	1/8	0.0367	Dasatinib
Potassium channel blocker	1/9	0.0367	Amiodarone
FGFR inhibitor	1/9	0.0367	Lenvatinib
Bcr-Abl kinase inhibitor	1/7	0.0367	Dasatinib
src inhibitor	1/7	0.0367	Dasatinib
angiogenesis inhibitor	1/6	0.0367	Tranilast
dihydrofolate reductase inhibitor	1/10	0.0383	Trimethoprim
progesterone receptor agonist	1/11	0.0397	Dienogest
FLT3 inhibitor	1/12	0.0410	Gilteritinib

**Table A2:** Selected drugs with upregulated target DEGs and their corresponding DrugBank information from scDrugPrio analysis to counteract treatment resistance in ESCC patient dataset.

Drug	DrugBank ID	Pharmacological Effect	Targeted Upregulated DEGs	Cell Type Clusters
Drospirenone	DB01395	PGR (agonist), NR3C2 (antagonist), AR (antagonist)	AR	B cells
Dienogest	DB09123	PGR (agonist), AR (antagonist)	AR	T cells
Enzacamene	DB11219	PGR (antagonist), AR (antagonist)	AR	Dendritic cells, T cells
Etanercept	DB00005	TNF (antibody)	TNF	Endothelial cells, Fibroblast, Cancer-associated fibroblast (CAF)
Adalimumab	DB00051	TNF (antibody)	TNF	Endothelial cells, Fibroblast, CAF
Infliximab	DB00065	TNF (inhibitor)	TNF	Endothelial cells, Fibroblast, CAF
Certolizumab pegol	DB08904	TNF (neutralizer)	TNF	Fibroblast
Amrinone	DB01427	PDE3A (inhibitor), PDE4B (inhibitor), TNF (inhibitor)	PDE4B	B cells
Amrinone	DB01427	PDE3A (inhibitor), PDE4B (inhibitor), TNF (inhibitor)	PDE3A, PDE4B, TNF	Fibroblast
Ambrisentan	DB06403	EDNRA (antagonist), EDNRB (antagonist)	EDNRB	Tumor-associated macrophages (TAM)
			EDNRA, EDNRB	B cells
			EDNRB	Endothelial cells
			EDNRA	Fibroblast
			EDNRB	Epithelial cells
			EDNRA	TAM
			EDNRA, EDNRB	Inflammatory CAF
			EDNRB	Epithelial cells
